# Supplementary material for: Advances in mixed cell deconvolution enable quantification of cell types in spatial transcriptomic data
Source: Nat Commun. 2022 Jan 19;13:385. doi: 10.1038/s41467-022-28020-5 (PMC8770643; doi:10.1038/s41467-022-28020-5)
Supplement: Supplementary file 1 — Supplementary Information [file 41467_2022_28020_MOESM1_ESM.pdf]

Supplementary Note 1

Commentary on the use of log-normal regression in deconvolution

Implicit in any deconvolution technique is a mean model, specifying how cell types' expression profiles add up to create a mixed profile; and a variance model, describing the noise between expected and observed expression. Traditional deconvolution techniques use a linear-scale mean model and a linear-scale variance model. SpatialDecon's use of log-normal regression retains this linear-scale mean model, but it replaces the linear-scale variance model with a log-scale variance model. In the following sections provide the rationale for this approach.

Rationale for the linear mean model used by SpatialDecon

SpatialDecon uses log-normal regression, which can be understood as a hybrid of linear-scale and log-scale approaches to the data. It models variance on the log-scale, for reasons explained above. But its mean model is on the linear scale; that is, it assumes total gene expression from mixed cell types is the sum of gene expression from individual cell types. Here, we justify this assumption.

One of the earliest gene expression deconvolution papers[1] ran constrained linear regression on log-transformed data, assuming the log-scale for both the mean and variance models. Others then argued that deconvolution should analyze linear-scale data[2], a conclusion the field has widely accepted. Below we rephrase the argument for a linear mean model in the nomenclature of this manuscript.

Say cell type k expresses gene j with mean level X_{jk}, and unspecified distribution. Then if a sample or spatial region contains β_k cells of type k, then those cells are expected to have a total of $X_{jk}\ \beta_k$ transcripts of gene j. If a sample contains multiple cell types, then the expected transcripts of gene j equals $\sum_k X_{jk} \beta_k$.

The above argument assumes only that each cell's expected expression of a gene is independent of the other cells in the sample. This assumption is overly simplistic, as cells react to their surroundings, but as a reasonable first-order approximation it has served the deconvolution literature since 2012. To our knowledge, no work yet has attempted to model interactions between cells while performing deconvolution.

In contrast, a log-scale mean model leads quickly to absurdity. The log-scale mean model assumes log(total transcripts of gene j) = log($X_{jk}\ \beta_k$), or equivalently, total transcripts of j = exp($X_{jk}\ \beta_k$). This model implies that if one T-cell has 10 transcripts of CD3E, then two T-cells will have 100 transcripts, three T-cells will have 1000 transcripts, and so on.

The above argues that a linear mean model accurately describes the number of transcripts present in a sample. But for a specific technology, we must consider not just the number of transcripts present, but the number of transcripts observed by the technology. Below we argue that this linear mean model of transcripts present applies equally well to the number of transcripts observed using spatial gene expression platforms.

Both Visium and GeoMx capture available transcripts with less-than-perfect efficiency. Define α as the rate at which transcripts that are present are observed by a platform. Then if the expected transcripts present in a sample is $\sum_k X_{jk}\beta_k$, the expected transcripts counted is simply $\alpha \sum_k X_{jk}\beta_k$. For the purposes of deconvolution, the α term can be framed as a uniform rescaling of X and therefore ignored.

The above argument assumes that the probability of a transcript being observed by these technologies is independent of the other transcripts present. Since neither technology has been shown to suffer substantial signal saturation, and since their sampling methods do not have mechanisms by which one gene can interfere with another's measurement, this assumption is reasonable.

Rationale for the log-scale variance model used by SpatialDecon

Deconvolution algorithms with linear-scale variance models solve extensions of the optimization problem ||y – Xβ||, where y is a sample's (linear-scale) expression vector, X is the cell profile matrix, β is the vector of cell types abundances, and ||*|| is the L2 norm operator. Implicit in this objective function are two assumptions: first, that all genes have comparable variance, and second, that error in gene expression is unskewed. If some genes were far more variable than the others, they would have more extreme residuals and would therefore exert undue influence on the deconvolution fit. And if genes were positively skewed, then positive residuals would be more likely, and deserving of less influence, than negative residuals of the same magnitude.

In contrast to classical deconvolution methods, SpatialDecon uses log-normal regression, which optimizes ||log(y) – log(Xβ)||. This model is motivated by the claim that linear-scale gene expression has extremely unequal variance and a strong tendency to positive skewness, and that log-transformed gene expression largely corrects these behaviors. Below, we demonstrate this claim.

A simple thought experiment provides intuition for the claim of unequal variance. Consider two genes, one with an average of 10 transcripts per sample, and one with an average of 10,000 transcripts per sample. The 10-transcript gene almost certainly has a standard deviation not much higher than 10: because negative expression levels are impossible, only with a distribution of primarily zeroes and an occasional very high expression level could it achieve a SD an order of magnitude higher than its mean. In contrast, the 10,000-transcript gene almost certainly has a high standard deviation: gene expression is controlled by diverse feedback loops, inhibitors and promoters, and it is hard to imagine this complex biological system controlling a gene's expression level at a level of precision like 10,000 +/- 10 counts. A more plausible scenario would be that both genes are controlled at +/- 10%. Technical noise in counting transcripts also contributes to unequal variance. If a given platform samples transcripts with some fixed probability p, then the observed counts have a binomial(n, p) distribution, where n is the number of transcripts available. Under this simplistic model, our 10-count gene would have a technical variance of 10p(1-p), while our 10000-count gene would have a technical variance of 10000p(1-p): 1000-fold higher.

Supplementary Figure 1 uses data from the TCGA LUAD (lung adenocarcinoma) RNAseq dataset to demonstrate gene expression's skewness and unequal variance on the linear-scale and its relative normality and consistent variance on the log-scale. Panel (a) shows the distribution of CD274 (PD-L1), a

gene with typical skewness. On the linear scale its skewness is 2.8; after log-transformation its skewness is 0.1 (the normal distribution has skewness of 0). Panel (b) shows the distribution of the skewness statistics from all genes in the transcriptome in TCGA LUAD. On the linear-scale, all but one of the 20243 genes measured was right-skewed, and 68% have extreme skewness > 2. On the log-scale, the average gene has skewness close to 0, and only 0.4% of genes have skewness outside of (-2, 2). Panel (c) demonstrates the unequal variance of gene expression, plotting each gene's SD against its mean. In linear-scale data, SD increases proportional to a gene's mean expression level, and the range of SDs spans $9.6 \cdot 10^{-3}$ to $1.9 \cdot 10^{5}$ (20004870-fold). In log-scale data, low-expression genes are only slightly more variable than high-expressors, and the range of SDs is only 16.5-fold.

Supplementary Figure 2 repeats the analysis of Supplementary Figure 1 using GeoMx data instead of TCGA. Again, we find skewness and unequal variance on the linear-scale and relative normality and consistent variance on the log-scale. Panel (a) shows the distribution of CD274 (PD-L1), a gene with typical skewness. On the linear scale its skewness is 1.2; after log-transformation its skewness is 0.4. Panel (b) shows the distribution of the skewness statistics from all genes in the transcriptome in TCGA LUAD. On the linear-scale, 1657 of 1700 genes are right-skewed, and 9% have extreme skewness > 2. On the log-scale, the average gene has skewness close to 0, and only 0.4% of genes have skewness outside of (-2, 2). Panel (c) demonstrates the unequal variance of gene expression, plotting each gene's SD against its mean. In linear-scale data, SD increases proportional to a gene's mean expression level, and the range of SDs spans 0.83 to 997 (1199-fold). In log-scale data, low-expression genes are only slightly more variable than high-expressors, and the range of SDs is only 10.5-fold.

Supplemental Figure 3 turns to healthy tissues. In a GeoMx dataset from a healthy kidney and a GeoMx dataset from a healthy pancreas, we compute genes' mean, standard deviation and skewness under the linear and log scales. In both these datasets, we again find that in the linear scale, high-expression genes have SDs up 1000-fold higher than low-expression genes, and we find that log-transformation greatly condenses the range of genes' standard deviations. We also see that linear-scale data is highly right-skewed, while on the log-scale genes' have skewness much closer to 0.

Specifically, in kidney, on the linear-scale, the smallest skewness from the 18695 is 21. On the log-scale, the average gene has skewness of 0.92, close to 0, and only 13% of genes have skewness outside of (-2, 2). In linear-scale data, SD increases proportional to a gene's mean expression level, and the range of SDs spans 2.14 to 10807 (5049-fold). In log-scale data, low-expression genes are only slightly more variable than high-expressors, and the range of SDs is only 9-fold.

In pancreas, on the linear-scale, the smallest skewness from the 18695 is 51. On the log-scale, the average gene has skewness close of 2.2. In linear-scale data, SD increases proportional to a gene's mean expression level, and the range of SDs spans 0.96 to 17460 (180756-fold). In log-scale data, low-expression genes are only slightly more variable than high-expressors, and the range of SDs is 100-fold (some genes had SDs close to 0).

We have seen in four datasets that log-transformation gives genes more consistent variance and less skewness. This conclusion motivates our use of log-normal regression, which seeks to minimize $||\log(y)-\log(X\beta)||$. In Figure 2 of the main text, we see this argument borne out: log-normal regression

outperforms linear methods like NNLS and v-SVR, which both suffer extreme influence from a small number of genes. DWLS, a least squares method that attempts to appropriately weight each gene, performs on par with log-normal regression, suggesting that the weakness of least-squares deconvolution lies in unequal variance and not in skewness.

Finally, a note on the role of biological and technical error in these considerations: Different genomics platforms measure gene expression with different chemistries, making each subject to idiosyncratic technical variance in measured gene expression. But in general, biological variability overwhelms technical variability; this being the case, we suggest that deconvolution algorithms must focus primarily on modelling biological variability, and that variance models designed around technical variability would miss the larger picture. To see that biological variability is much larger than technical variability in spatial genomics platforms, see reproducibility studies for GeoMx[3] and Spatial Transcriptomics[4].

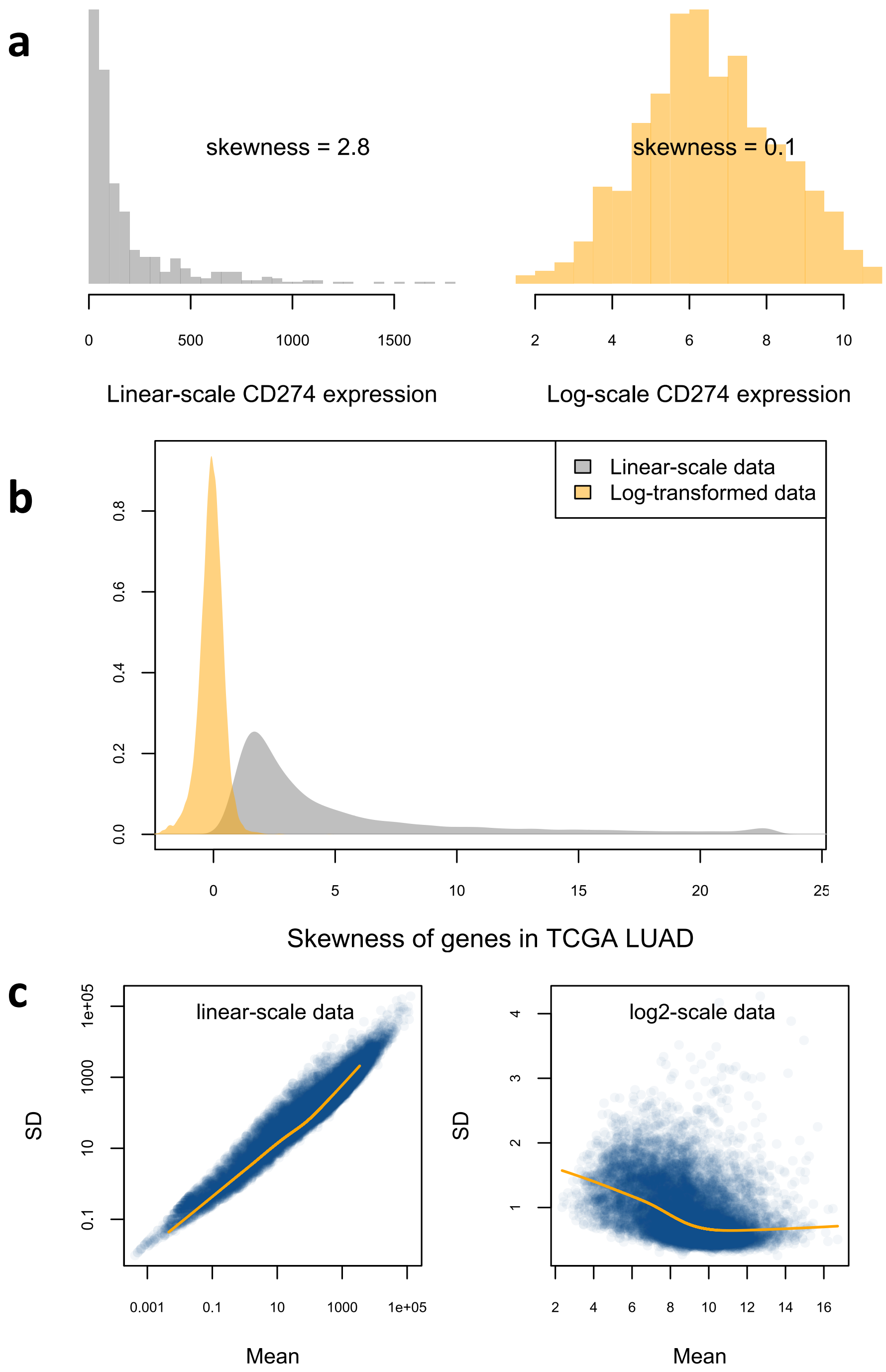

Supplementary Figure 1: skewness and unequal variance of TCGA gene expression data. *All figures are generated from the TCGA LUAD dataset.* ***a.*** *Histograms of CD274 (PD-L1) expression on the log and linear scale.* ***b.*** *Distribution of skewness statistics calculated for each of 20531 genes across the TCGA LUAD samples. Grey: skewness of linear-scale genes. Orange: skewness of log-scale genes.* ***c.*** *Genes' mean and standard deviation, calculated from linear-scale and from log-scale data.*

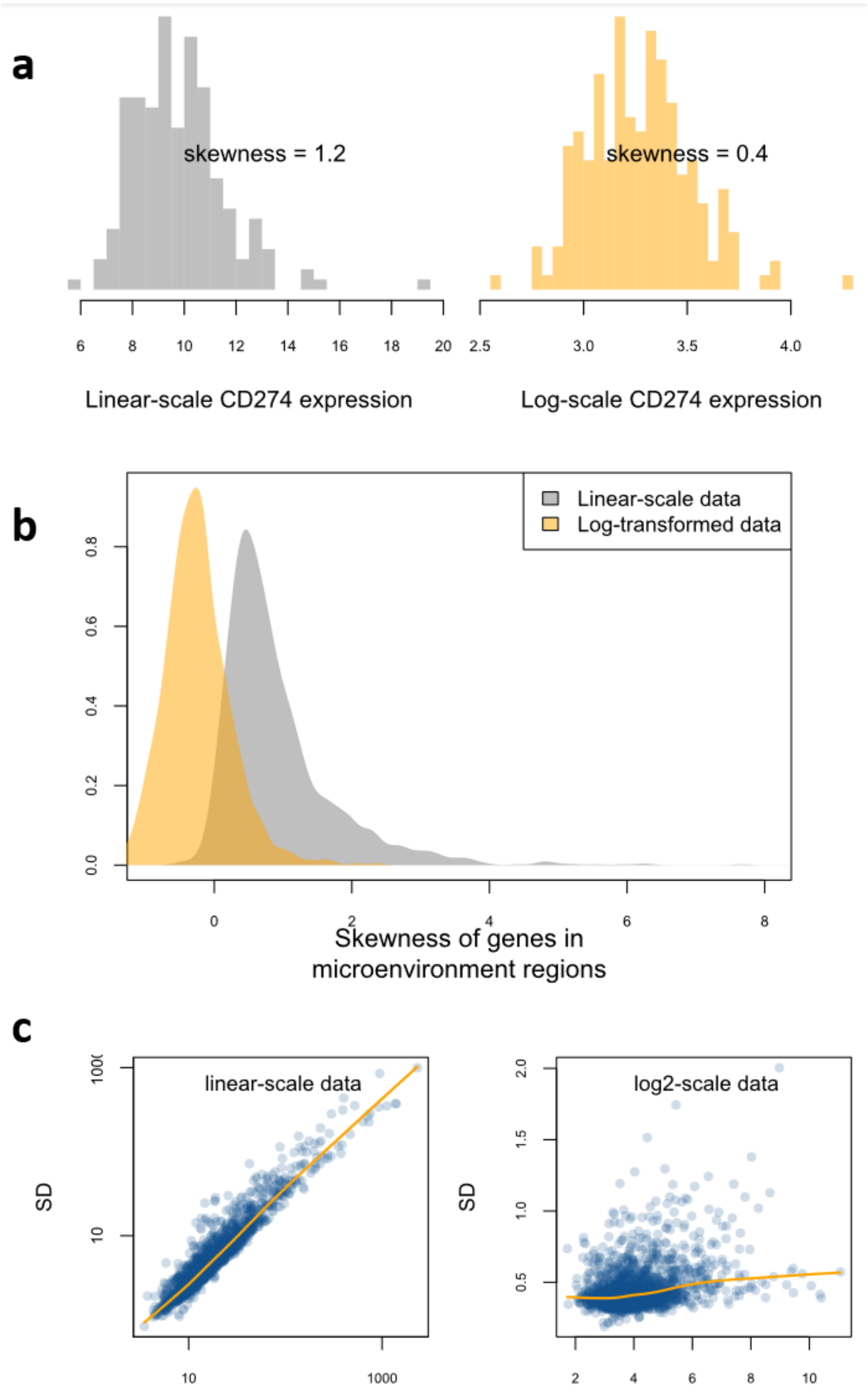

Supplementary Figure 2: skewness and unequal variance of GeoMx gene expression data in cancer. *All figures are generated from the microenvironment regions of the NSCLC tumor analyzed in Figures 5 and 6. Tumor regions were excluded due to concerns that the profound differences between tumor and microenvironment regions would cloud interpretation of results.* ***a.*** *Histograms of CD274 (PD-L1) expression on the log and linear scale.* ***b.*** *Distribution of skewness statistics calculated for each of 1700 genes. Grey: skewness of linear-scale genes. Orange: skewness of log-scale genes.* ***c.*** *Genes' mean and standard deviation, calculated from linear-scale and from log-scale data.*

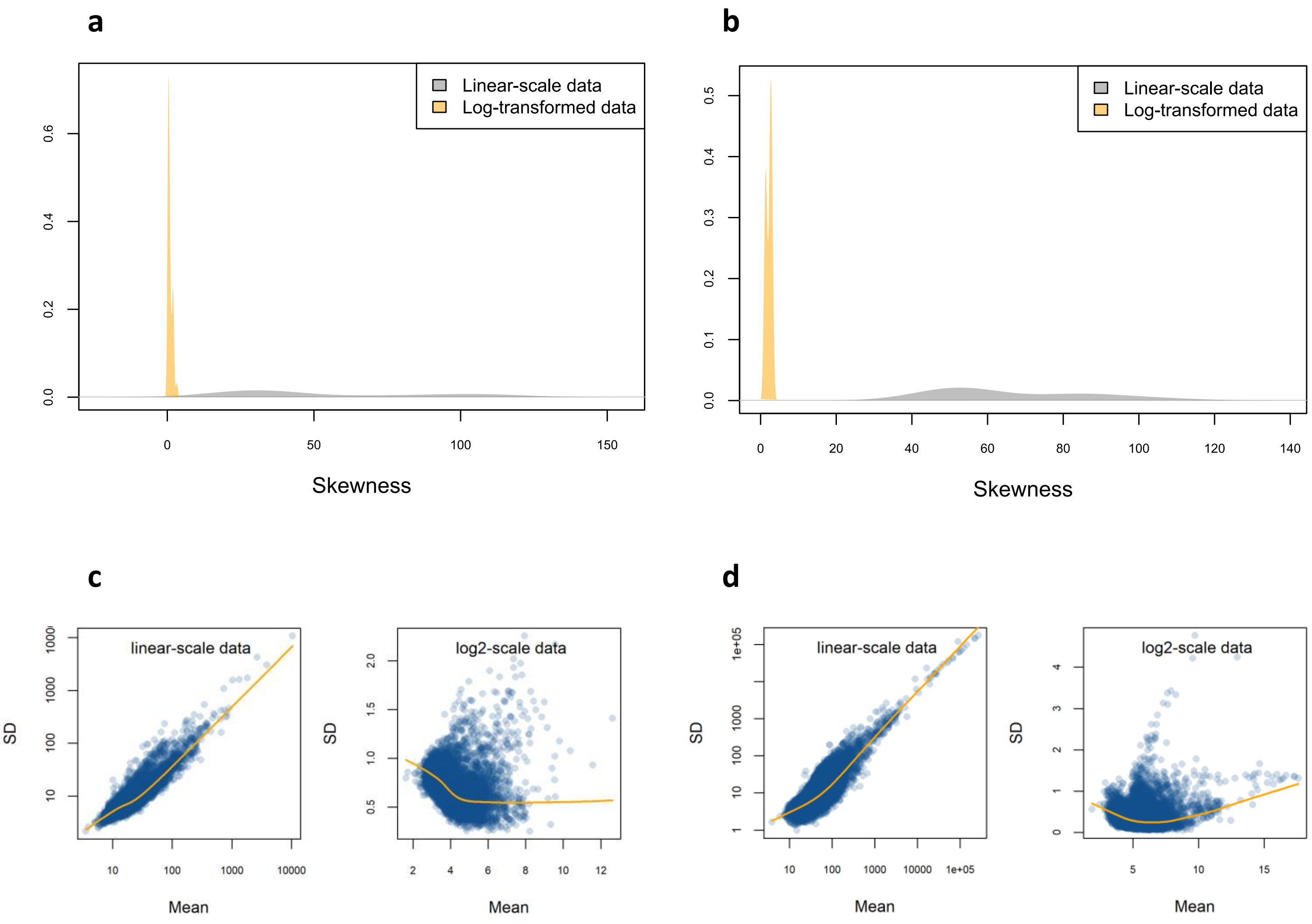

Supplementary Figure 3: skewness and unequal variance of GeoMx gene expression data in healthy tissues. *GeoMx Whole Transcriptome Atlas data was collected from 39 regions sampled from a healthy kidney and 10 regions sampled from a healthy pancreas.* ***a, b.*** *Distribution of skewness statistics calculated for each of 18695 genes in kidney and pancreas, respectively. Grey: skewness of linear-scale genes. Orange: skewness of log-scale genes.* ***c, d.*** *From kidney and pancreas, respectively: genes' mean and standard deviation, calculated from linear-scale and from log-scale data.*

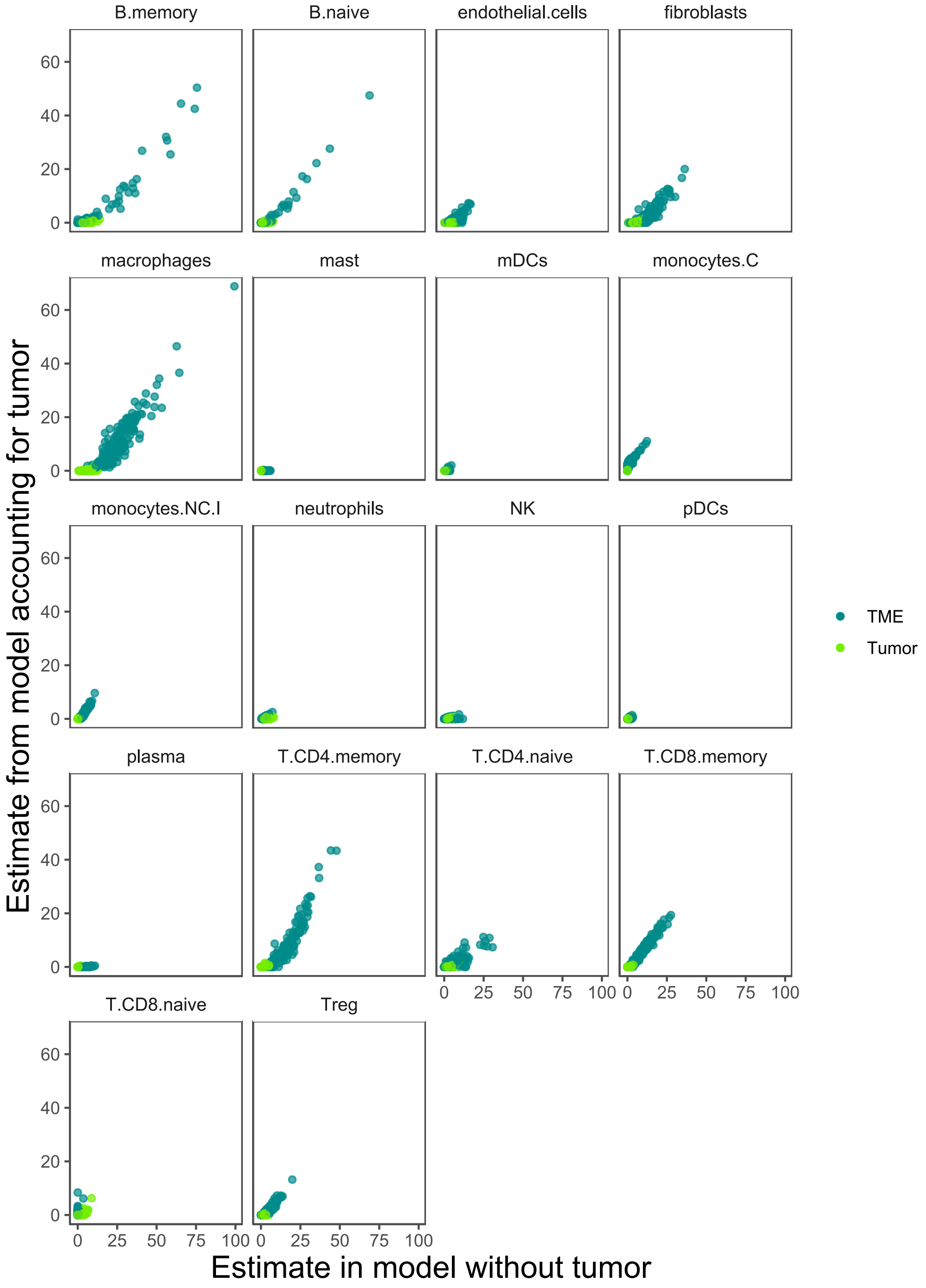

Supplementary Figure 4: Immune and stroma cell abundance estimates from segments of a NSCLC tumor, with and without modelling tumor-specific expression. Horizontal axis: *Estimates from deconvolution using only stroma cell profiles.* ***Vertical axis:*** *Estimated from deconvolution using both stroma cell profiles and 10 tumor cell profiles derived from pure tumor segments.*

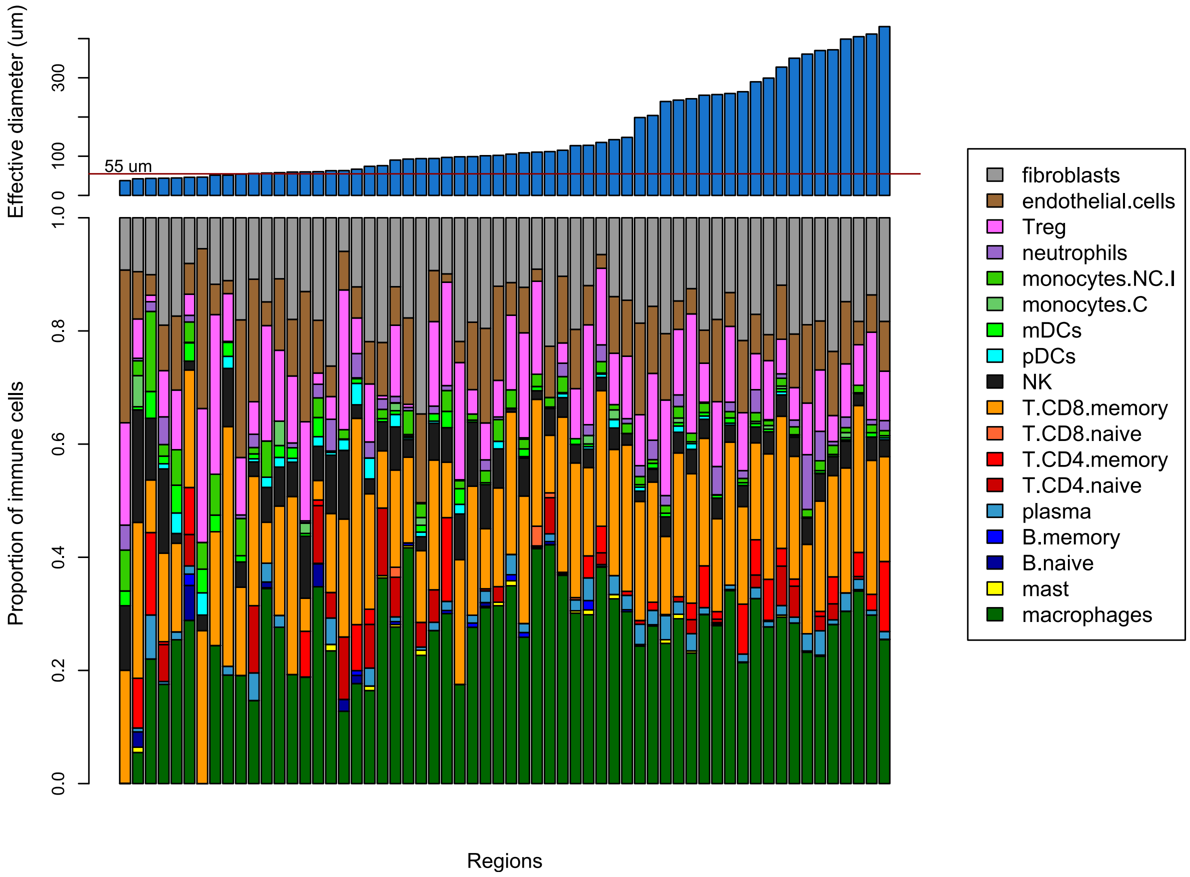

Supplementary Figure 5: Consistency of results from small to large regions. *In a colorectal tumor, microenvironment (PanCK-) regions were profiled with GeoMx Cancer Transcriptome Atlas. Region areas ranged from 1119µm^2 (equivalent to a spot of diameter 37.7µm) to 145633µm^2 (equivalent to a diameter of 430.6µm).*

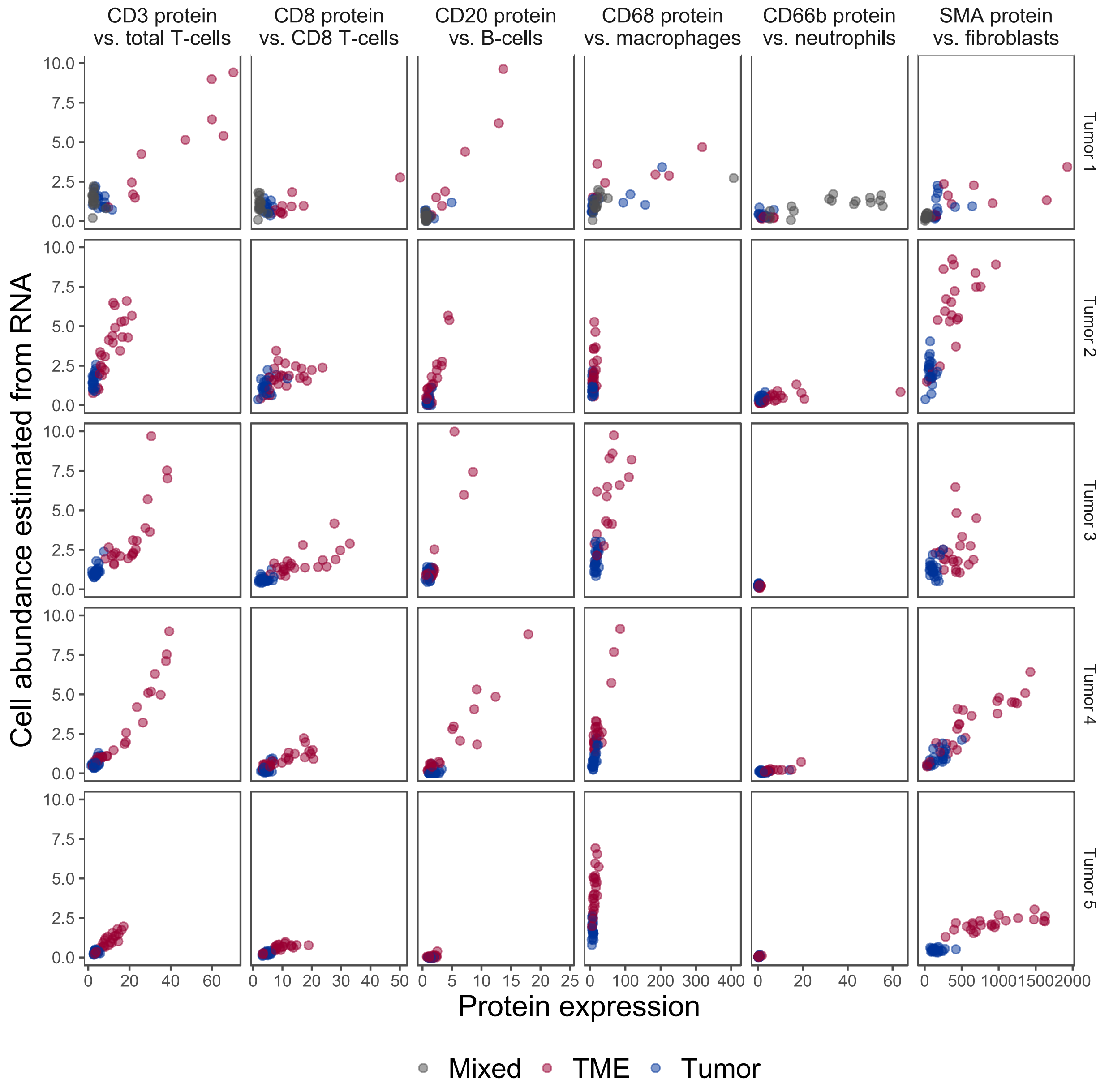

Supplementary Figure 6: Deconvolution performance using granularly-defined cell types. *Expression of marker proteins (horizontal axis) against cell abundance estimates from gene expression deconvolution (vertical axis). Abundance scores from related cell types, e.g. 9 macrophage subsets, have been added together. Each column of panels shows results from a single protein/cell pair; each row shows results from a different lung tumor. Tumor segments are shown in blue, microenvironment segments in red.*

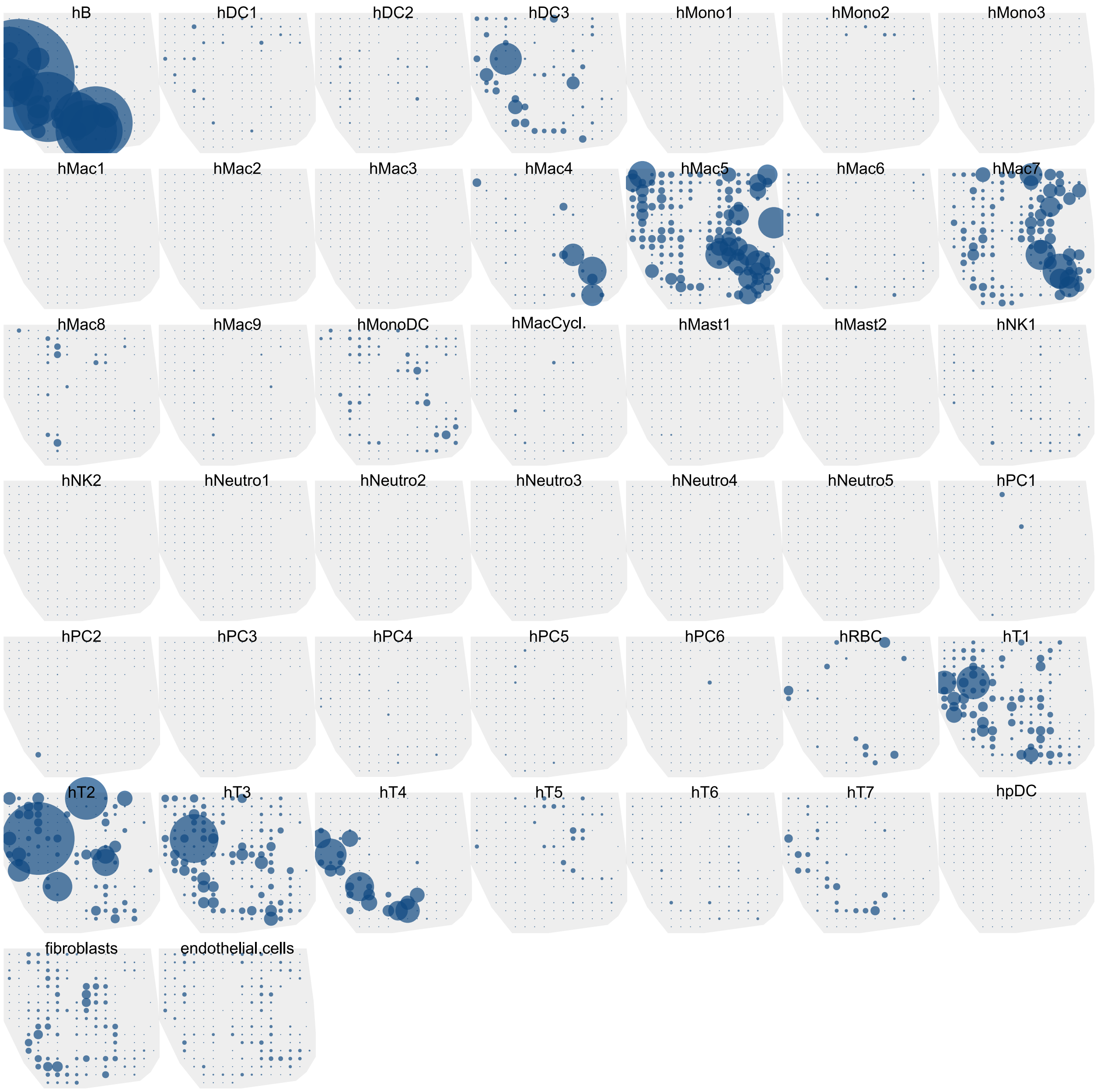

Supplementary Figure 7: Deconvolution results using granularly-defined cell types. *Point size corresponds to abundance score.*

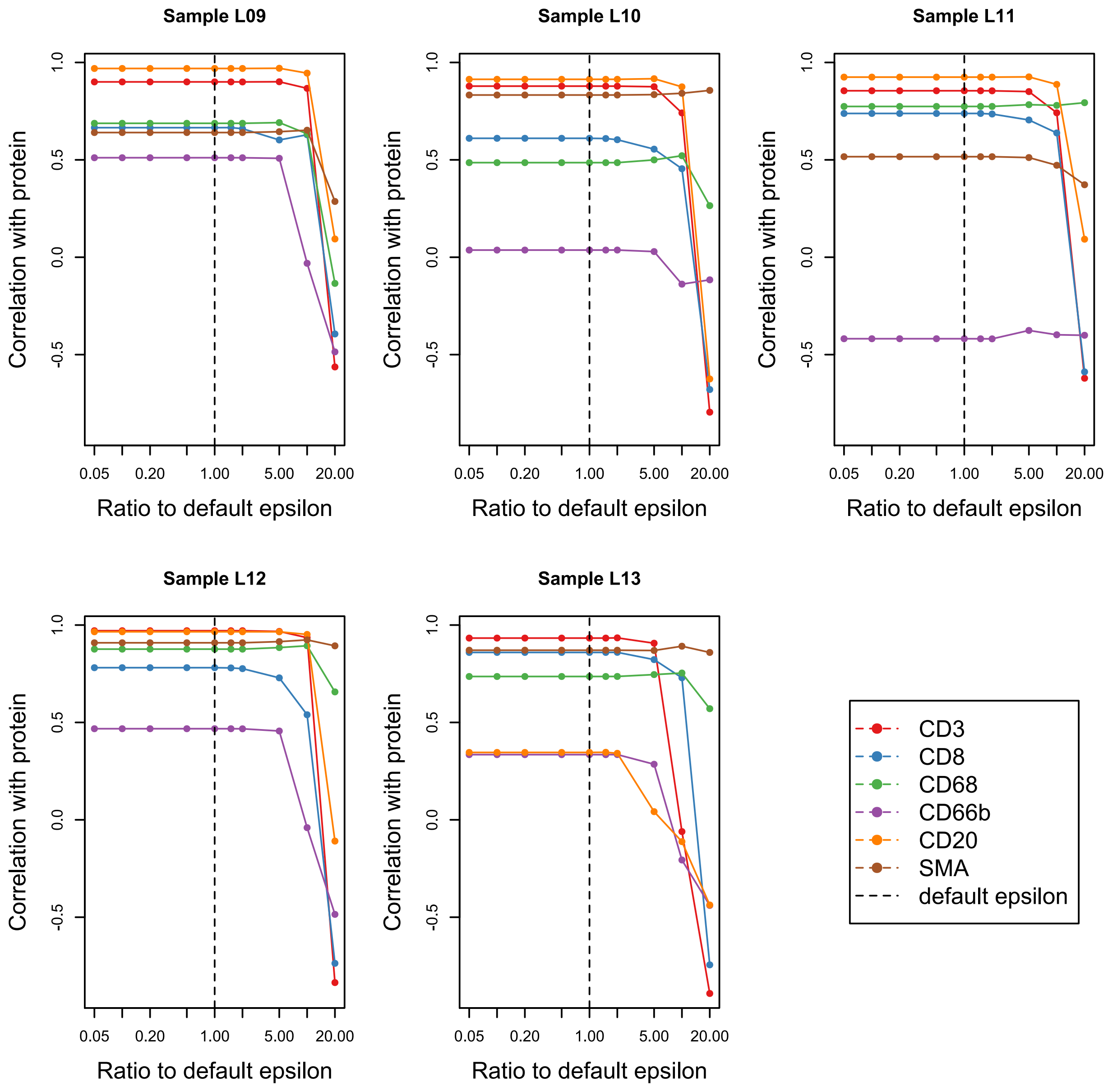

Supplementary Figure 8: Impact of epsilon on Algorithm 1. *Epsilon is the lower threshold used by Algorithm 1 to avoid log-transforming zeroes. For a range of epsilons, Algorithm 1 was applied to the benchmarking data of Figure 4. For each cell type/protein pair and each tissue, the correlation between Algorithm 1's results and the corresponding marker protein is shown. The default value of epsilon is shown with a dashed line. For all tissues and for all cell type/protein pairs, there is no change in accuracy for any epsilon up to double the default value, confirming that Algorithm 1 is not sensitive to reasonable choices of epsilon.*

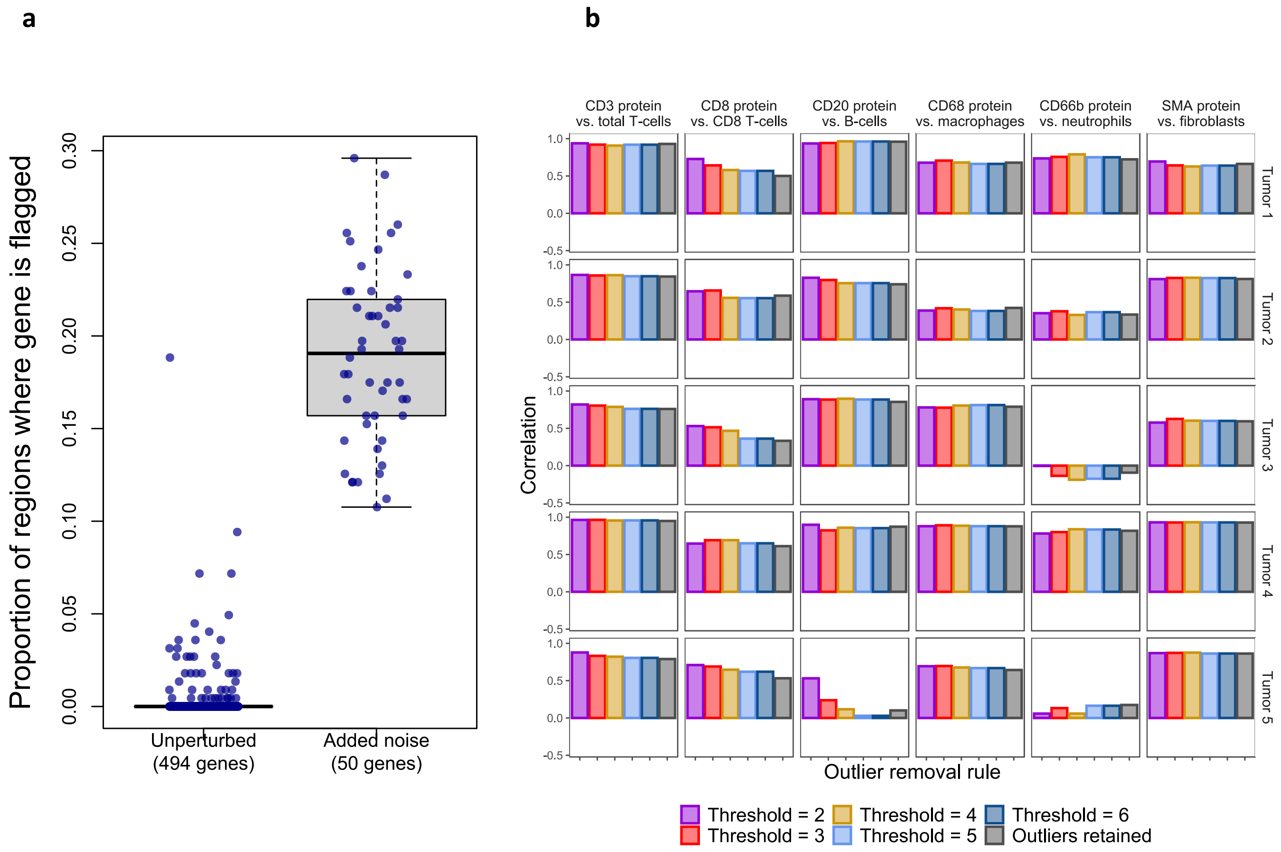

Supplementary Figure 9: Behavior of outlier detection procedure in NSCLC benchmarking data. *In the benchmarking dataset of Figure 4, the normalized data from 50 genes was multiplied by log-normally distributed scaling factors with a log2-scale SD of 3.* ***a.*** *Results of SpatialDecon's outlier-removal mechanism. For each gene, vertical position shows the proportion of regions in which the gene was removed as an outlier. Boxplot centers show median values, box limits show 0.25 and 0.75 quantiles, whiskers extent to the most extreme point with a distance from the box less than 1.5 times the interquartile range of the data.* ***b.*** *Impact of outlier removal on deconvolution performance. In the same perturbed dataset, SpatialDecon was run with outlier removal with and without outlier removal. Outlier removal was performed under a range of thresholds, calculated on the scale of log2 fold-change between observed and fitted values. For each tissue and each cell type / marker protein pair, the correlation between SpatialDecon and the marker protein is shown. Removing outliers improves average correlation from 0.65 to 0.69 under the default threshold of 3 (paired t-test two-sided p = 0.004).*

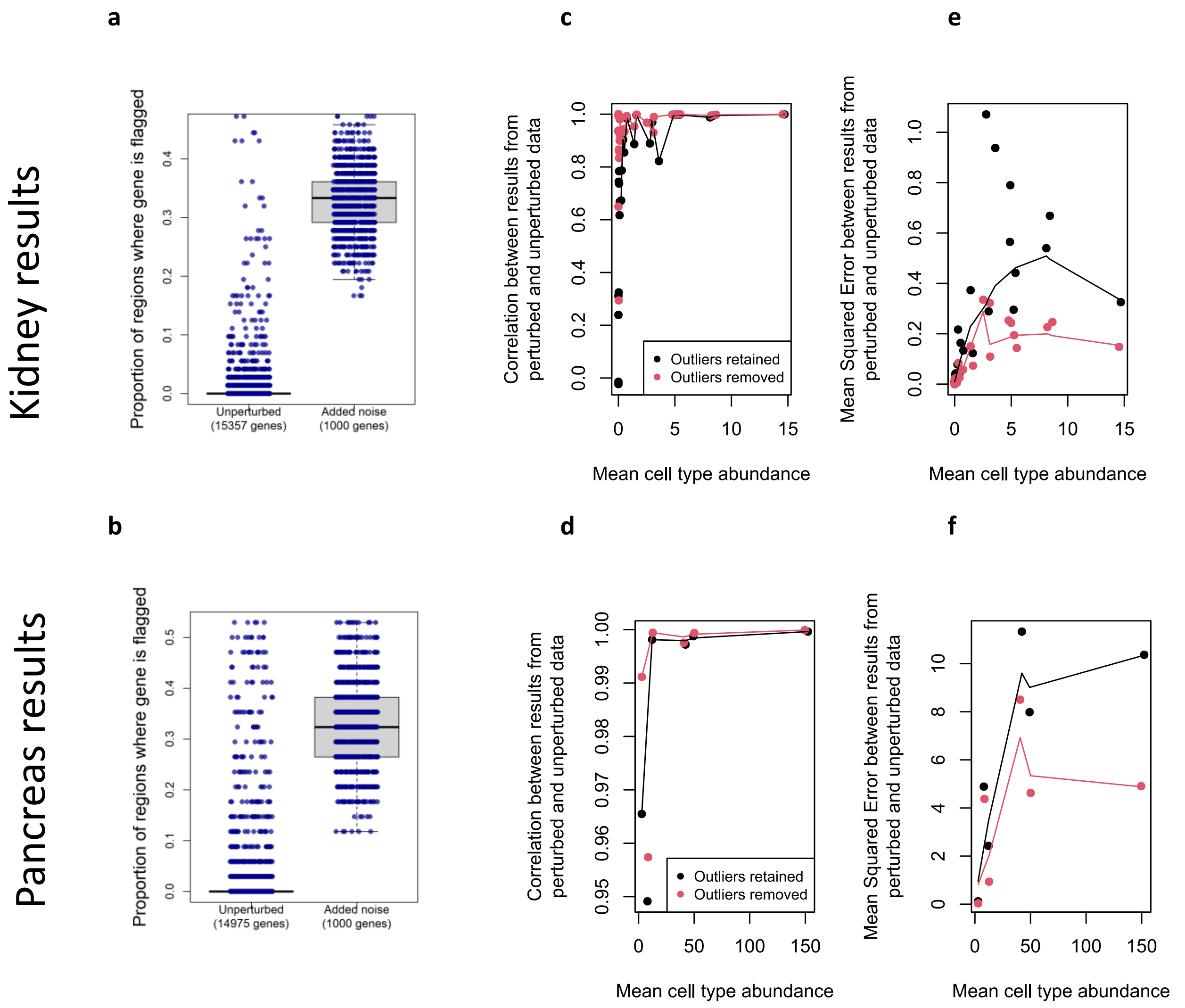

Supplementary Figure 10: Performance of outlier detection in healthy tissues. *GeoMx Whole Transcriptome Atlas data was collected from 39 regions sampled from a healthy kidney and 10 regions sampled from a healthy pancreas. The normalized data from 1000 genes was then multiplied by log-normally distributed scaling factors to create a perturbed dataset. SpatialDecon was performed on the original and the perturbed datasets, first retaining outliers and then removing outliers. "Outlier-retained" results were compared across the original and perturbed datasets, as were "outlier-removed" results.* ***a,b.*** *Results of SpatialDecon's outlier-removal mechanism in kidney and pancreas, respectively. For each gene, vertical position shows the proportion of regions in which the gene was removed as an outlier. The genes with added noise were flagged at higher rates. Boxplot centers show median values, box limits show 0.25 and 0.75 quantiles, whiskers extent to the most extreme point with a distance from the box less than 1.5 times the interquartile range of the data.* ***c,d.*** *From kidney and pancreas respectively, correlation between deconvolution results from original and perturbed data. Each point shows results for a single gene. Red and black dots show results using and omitting outlier detection.* ***e,f.*** *From kidney and pancreas respectively, mean squared error between deconvolution results from original and perturbed data.*

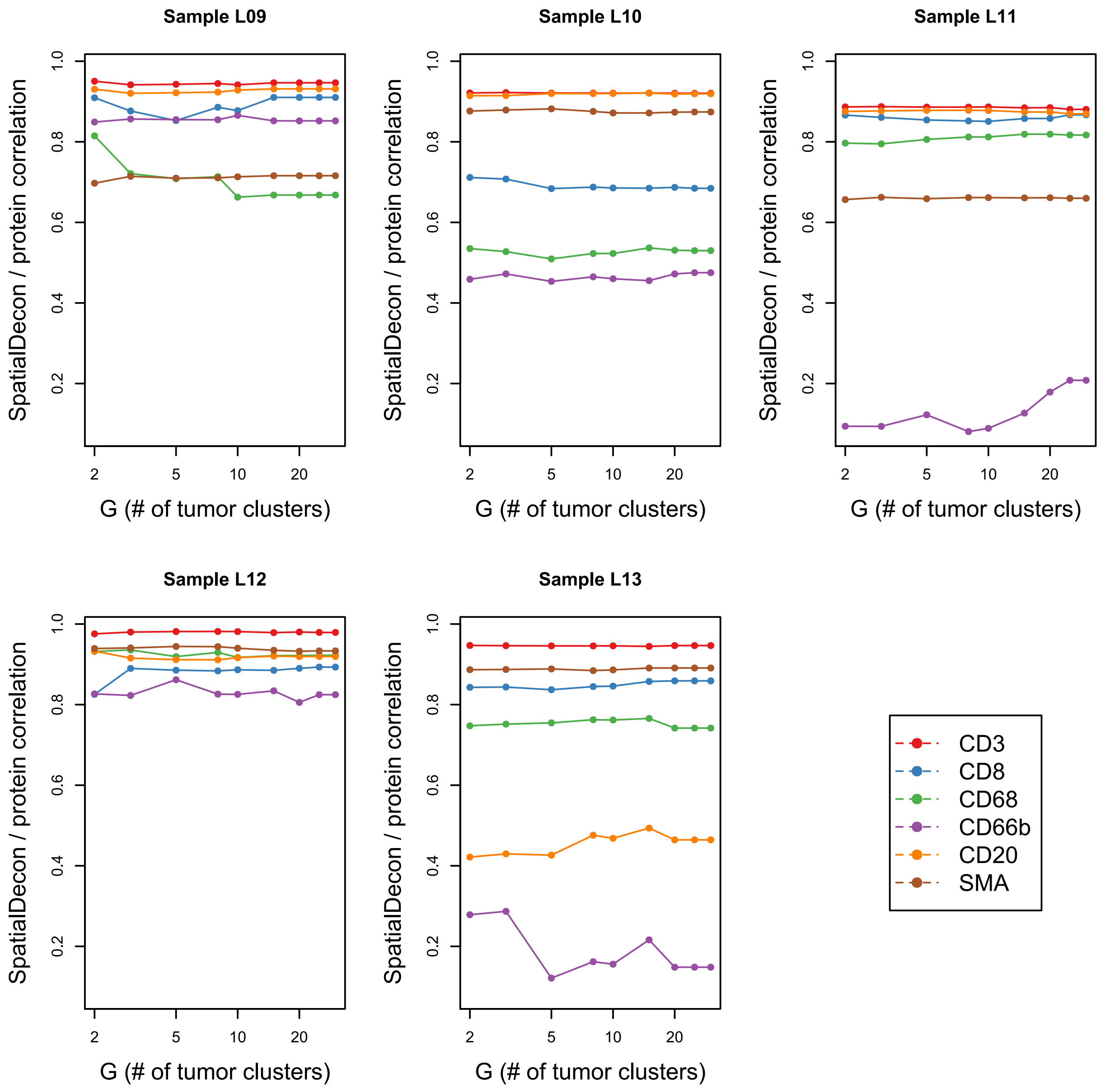

Supplementary Figure 11: Impact of G, the number of inferred tumor clusters, on performance. *SpatialDecon can use regions identified as pure tumor to infer tumor-specific expression profiles, which can then be appended to the SafeTME matrix for improved accuracy. The number of tumor profiles inferred is a user-facing argument, "G". Here, we explore the impact of this argument on deconvolution performance. For each tissue in the benchmarking dataset of Figure 4, SpatialDecon was run using a range of values of G. Correlation between marker proteins and SpatialDecon results are shown for each tissue, cell type, and choice of G. G has little impact on accuracy.*

a

b

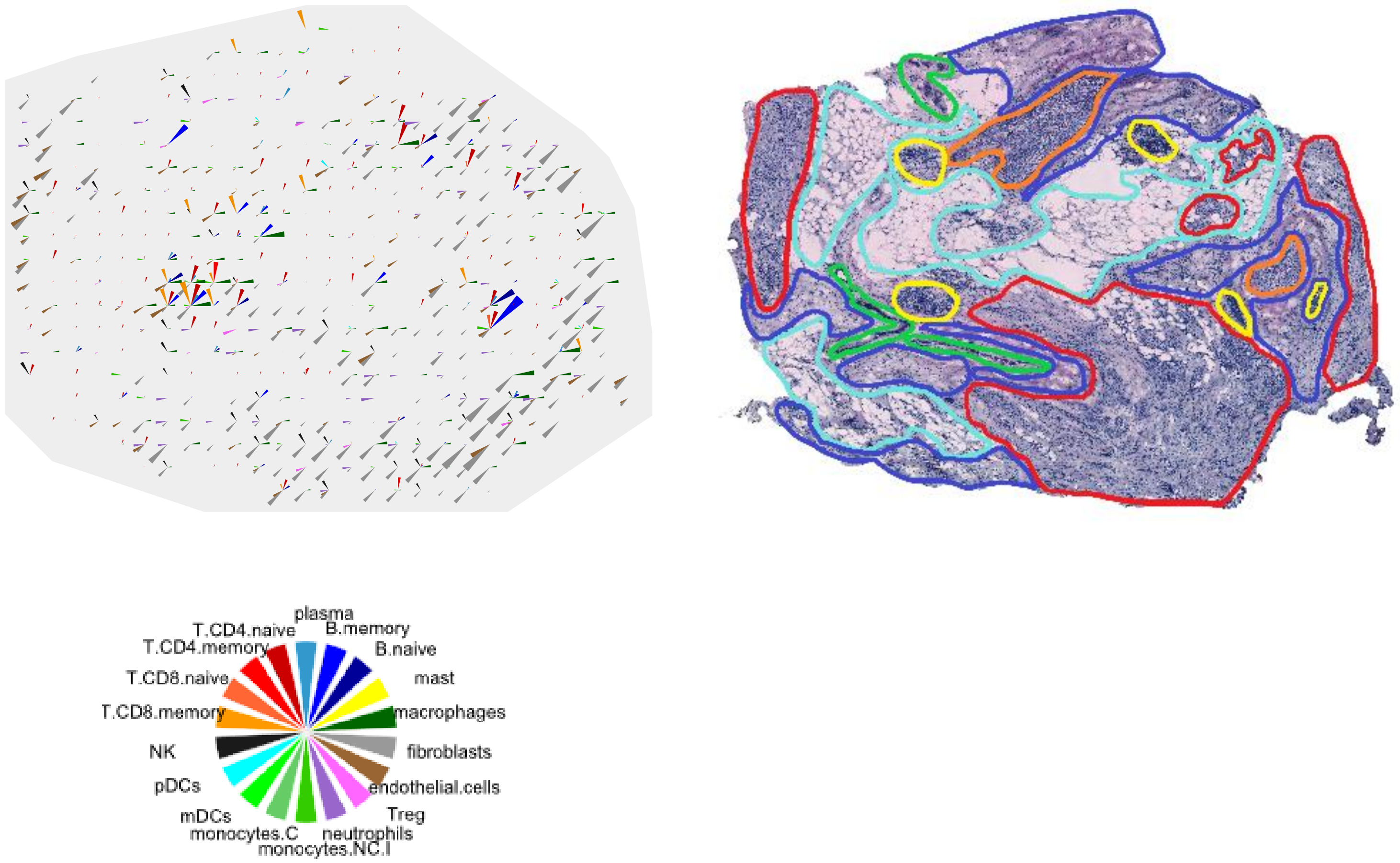

Supplementary Figure 12: Application of SpatialDecon to Spatial Transcriptomics data. *SpatialDecon using the SafeTME matrix was applied to Spatial Transcriptomics data from a breast tumor[5] ("Tissue G" from Andersson et al. 2020). Per our recommendations, a background level of 0.01 counts was specified. The 50 regions with the lowest ratios of SafeTME gene counts over total gene counts were designated to SpatialDecon as "pure tumor".* ***a****. Cell type abundance estimates from SpatialDecon.* ***b****. From Andersson et al. (2020): pathologist's annotations, with yellow encircling inflammatory cells. The pathologist-circled inflammatory cell regions contain high SpatialDecon abundance scores for B-cells and T-cells.*

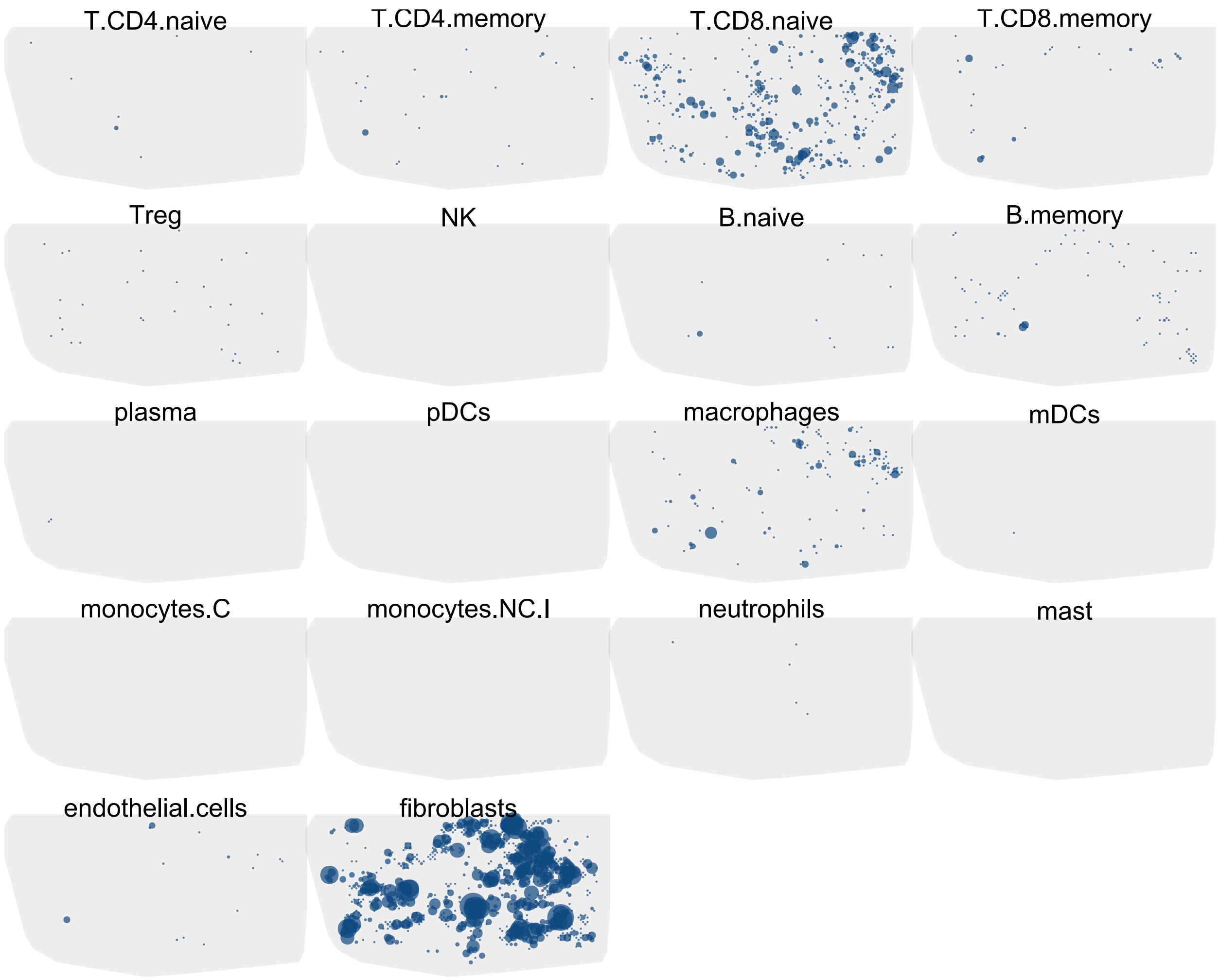

Supplementary Figure 13: Application of SpatialDecon to Visium data. *SpatialDecon using the SafeTME matrix was applied to Spatial Transcriptomics data from an ovarian tumor (from https://support.10xgenomics.com/spatial-gene-expression/datasets/1.2.0/Parent_Visium_Human_OvarianCancer). Per our recommendations, a background level of 0.01 counts was specified. The 100 regions with the lowest ratios of SafeTME gene counts over total gene counts were designated to SpatialDecon as "pure tumor". This tumor's immune infiltrate is dominated by naïve CD8 T-cells and macrophages, both of which are diffused widely across the tumor. In addition, fibroblasts are abundant. These results are consistent with reports on the content of the tumor microenvironment in Ovarian cancer*[6,7,8].

References

1. Shen-Orr, Shai S., et al. Cell type–specific gene expression differences in complex tissues. *Nature methods* **7(4)**, 287-289 (2010).
2. Zhong, Y. & Liu, Z. Gene expression deconvolution in linear space. *Nature methods* **9(1),** 8-9 (2012).
3. Merritt, C. R., Ong, G. T., Church, S. E., Barker, K., Danaher, P., Geiss, G., ... & Beechem, J. M. (2020). Multiplex digital spatial profiling of proteins and RNA in fixed tissue. *Nature Biotechnology*, ***38*(5)**, 586-599.
4. Stark, R., Grzelak, M., & Hadfield, J. (2019). RNA sequencing: the teenage years. *Nature Reviews Genetics*, ***20*(11)**, 631-656.
5. Andersson, Alma E.V., et al. "Spatial deconvolution of HER2-positive breast tumors reveals novel intercellular relationships." Preprint at https://www.biorxiv.org/content/10.1101/2020.07.14.200600v1 (2020).
6. Fujisawa, M., Moh-Moh-Aung, A., Zeng, Z., Yoshimura, T., Wani, Y., & Matsukawa, A. (2018). Ovarian stromal cells as a source of cancer-associated fibroblasts in human epithelial ovarian cancer: a histopathological study. *PLoS One*, ***13*(10),** e0205494.
7. Kawamura, K., Komohara, Y., Takaishi, K., Katabuchi, H., & Takeya, M. (2009). Detection of M2 macrophages and colony-stimulating factor 1 expression in serous and mucinous ovarian epithelial tumors. *Pathology international*, ***59*(5),** 300-305.
8. Wang, W., Zou, W., & Liu, J. R. (2018). Tumor-infiltrating T cells in epithelial ovarian cancer: predictors of prognosis and biological basis of immunotherapy. *Gynecologic oncology*, ***151*(1)**, 1.
