# Supplementary material for: Advances in mixed cell deconvolution enable quantification of cell types in spatial transcriptomic data
Source: Nat Commun. 2022 Jan 19;13:385. doi: 10.1038/s41467-022-28020-5 (PMC8770643; doi:10.1038/s41467-022-28020-5)
Supplement: Supplementary file 8 — Reporting Summary [file 41467_2022_28020_MOESM8_ESM.pdf]

nature research

Corresponding author(s): Patrick Danaher

Last updated by author(s): Oct 23, 2020

Reporting Summary

Nature Research wishes to improve the reproducibility of the work that we publish. This form provides structure for consistency and transparency in reporting. For further information on Nature Research policies, see our Editorial Policies and the Editorial Policy Checklist.

Statistics

For all statistical analyses, confirm that the following items are present in the figure legend, table legend, main text, or Methods section.

n/a	Confirmed	
☐	☒	The exact sample size (*n*) for each experimental group/condition, given as a discrete number and unit of measurement
☒	☐	A statement on whether measurements were taken from distinct samples or whether the same sample was measured repeatedly
☐	☒	The statistical test(s) used AND whether they are one- or two-sided<br>*Only common tests should be described solely by name; describe more complex techniques in the Methods section.*
☒	☐	A description of all covariates tested
☒	☐	A description of any assumptions or corrections, such as tests of normality and adjustment for multiple comparisons
☐	☒	A full description of the statistical parameters including central tendency (e.g. means) or other basic estimates (e.g. regression coefficient) AND variation (e.g. standard deviation) or associated estimates of uncertainty (e.g. confidence intervals)
☐	☒	For null hypothesis testing, the test statistic (e.g. *F*, *t*, *r*) with confidence intervals, effect sizes, degrees of freedom and *P* value noted<br>*Give P values as exact values whenever suitable.*
☒	☐	For Bayesian analysis, information on the choice of priors and Markov chain Monte Carlo settings
☒	☐	For hierarchical and complex designs, identification of the appropriate level for tests and full reporting of outcomes
☐	☒	Estimates of effect sizes (e.g. Cohen's *d*, Pearson's *r*), indicating how they were calculated

Our web collection on statistics for biologists contains articles on many of the points above.

Software and code

Policy information about availability of computer code

Data collection	GeoMx RNA data was collected using development pipelines of the commercial GeoMx Data Analysis software (v0). GeoMx protein data was collected using nSolver 4.0.
Data analysis	All data analysis was performed using R, version 4.0. Complete data analysis scripts can be found at https://github.com/Nanostring-Biostats/SpatialDecon-manuscript-analyses. The following R libraries were used: SpatialDecon 1.3.0 (https://github.com/Nanostring-Biostats/SpatialDecon), NormqPCR 1.34.0 (geNorm algorithm), e1071 1.7-9, logNormReg 0.3.0, pheatmap 1.0.12.

For manuscripts utilizing custom algorithms or software that are central to the research but not yet described in published literature, software must be made available to editors and reviewers. We strongly encourage code deposition in a community repository (e.g. GitHub). See the Nature Research guidelines for submitting code & software for further information.

Data

Policy information about availability of data

All manuscripts must include a data availability statement. This statement should provide the following information, where applicable:
- Accession codes, unique identifiers, or web links for publicly available datasets
- A list of figures that have associated raw data
- A description of any restrictions on data availability

All data from this study is available online without restriction. The library of cell profile matrices is available at https://github.com/Nanostring-Biostats/CellProfileLibrary. The in-situ benchmarking dataset is available at https://github.com/Nanostring-Biostats/ImmuneDeconBenchmark. The data used to produce this manuscript’s results are available at https://github.com/Nanostring-Biostats/SpatialDecon-manuscript-analyses. The raw GeoMx gene expression datasets generated in this study are available in the Gene Expression Omnibus under accession numbers GSE174743 [https://www.ncbi.nlm.nih.gov/geo/query/acc.cgi?acc=GSE174743] (benchmarking data from 5 NSCLC tumors from Figure 4), GSE174746 [https://www.ncbi.nlm.nih.gov/geo/query/acc.cgi?acc=GSE174746] (cell

pellet array from Figure 2), and GSE174749 [https://www.ncbi.nlm.nih.gov/geo/query/acc.cgi?acc=GSE174749] (NSCLC tumor from Figures 5 and 6). The datasets used to derive the SafeTME matrix are available in the Gene Expression Omnibus under accession numbers GSE127465 [https://www.ncbi.nlm.nih.gov/geo/query/acc.cgi?acc=GSE127465] (lung tumor scRNA-seq), GSE107011 [https://www.ncbi.nlm.nih.gov/geo/query/acc.cgi?acc=GSE107011] (sorted PBMCs RNAseq), and GSE111907 [https://www.ncbi.nlm.nih.gov/geo/query/acc.cgi?acc=GSE111907] (stroma cells RNAseq).
Source data are provided with this paper.
TCGA data was accessed through https://gdac.broadinstitute.org/. The HER2+ breast cancer dataset was accessed at https://github.com/almaan/her2st. The ovarian cancer dataset was accessed at https://www.10xgenomics.com/resources/datasets.

Field-specific reporting

Please select the one below that is the best fit for your research. If you are not sure, read the appropriate sections before making your selection.

☒ Life sciences ☐ Behavioural & social sciences ☐ Ecological, evolutionary & environmental sciences

For a reference copy of the document with all sections, see nature.com/documents/nr-reporting-summary-flat.pdf

Life sciences study design

All studies must disclose on these points even when the disclosure is negative.

Sample size

Sample sizes were chosen as a tradeoff between cost and the expected amount of data needed to show trends and descriptive patterns. For Figure 2, no sample size calculation was performed; however, the chosen sample size comfortably exceeded what was needed for trends to appear unambiguously, as supported the the small p-values attained in the analysis around Figure 2. Figure 3 used TCGA and so sample size was not under our control. For Figure 4, no sample size calculation was performed; rather, we ran as many samples as time and resources allowed. The chosen sample size was sufficient to attain statistical significance when comparing SpatialDecon's performance to other methods. For Figures 5&6, which contain purely descriptive analyses, the sample size (number of ROIs) was chosen to be as large as possible subject to constraints imposed by the GeoMx's data collection capability.

Data exclusions

In the serial section analysis (Figure 4), 5 AOIs of RNA data and 13 AOIs of protein data were discarded based on low overall signal. Per standard practice, the threshold for declaring "low signal" was chosen to exclude AOIs with outlier low signal. (GeoMx experiments vary in their overall signal levels, so prespecifying a cutoff is usually ill-advised.) This threshold was selected before the rest of the analysis was performed.
In the analysis for Figures 5&6, Three PanCK+ regions with outlier low signal strength were removed from the analysis, again using a cutoff chosen by looking at the distribution of signal levels.

Replication

As spatial gene expression experiments are laborious and expensive, each dataset in this study was only collected once.

Randomization

NA: This was a purely observational study with no experimental condition that could be randomized.

Blinding

NA: This study has no groups of samples being compared to each other, so blinding is not applicable.

Reporting for specific materials, systems and methods

We require information from authors about some types of materials, experimental systems and methods used in many studies. Here, indicate whether each material, system or method listed is relevant to your study. If you are not sure if a list item applies to your research, read the appropriate section before selecting a response.

Materials & experimental systems

n/a	Involved in the study
☐	☒ Antibodies
☐	☒ Eukaryotic cell lines
☒	☐ Palaeontology and archaeology
☒	☐ Animals and other organisms
☐	☒ Human research participants
☒	☐ Clinical data
☒	☐ Dual use research of concern

Methods

n/a	Involved in the study
☒	☐ ChIP-seq
☒	☐ Flow cytometry
☒	☐ MRI-based neuroimaging

Antibodies

Antibodies used

All antibodies used are part of the NanoString GeoMx human immuno-oncology protein panel, a commercial product. Visualization antibodies include CD3-647 at 1:400 (Abcam, ab196147), CD45-594 at 1:40 (NanoString Technologies), PanCK-532 at 1:40 (NanoString Technologies), and SYTO 13 (Thermo Scientific S7575) at 1:10.
For the GeoMx protein assay, the primary antibody mix was made by combining the detection antibody modules (NanoString

	Technologies) at 1:25 and the visualization markers in Buffer W. The NSCLC were visualized with CD3-647 at 1:400 (Abcam, ab196147), CD45-594 at 1:40 (NanoString Technologies) and PanCK-532 at 1:40 (NanoString Technologies).
Validation	Validation of the antibodies in the GeoMx panel involved a suite of experiments and is described by the manufacturer here: https://www.nanostring.com/wp-content/uploads/2021/08/WP_GeoMx_Antibody_Validation_White_Paper.pdf

Eukaryotic cell lines

Policy information about cell lines

Cell line source(s)	We used HEK-293T and CCRF-CEM, sourced from Acepix Biosciences, Inc.
Authentication	No authentication of cell lines was performed, because our experiment only required that the two cell lines have distinct expression patterns and made no biological interpretation based on the cell lines' identities.
Mycoplasma contamination	Cell lines were not tested for contamination.
Commonly misidentified lines (See ICLAC register)	Neither of the cell lines we used is commonly misidentified.

Human research participants

Policy information about studies involving human research participants

Population characteristics	We obtained FFPE tumor samples from a commercial tissue vendor, ProteoGenex. Patient ages ranged from 66-77. There were 4 female and 1 male patients. Grades were 1-3, and stages spanned IIB-IIIA. Tumor sizes spanned from 4x3x3 to 14x10x8 cm.
Recruitment	Information on recruitment of these patients is not available from the vendor, but out study does not use any clinical data or make any comparisons between patients.
Ethics oversight	ProteoGenex collected these samples under their usual ethics protocols: https://www.proteogenex.com/about-us/ethics-policy/

Note that full information on the approval of the study protocol must also be provided in the manuscript.
